# Do Protocadherins Show Prognostic Value in the Carcinogenesis of Human Malignant Neoplasms? Systematic Review and Meta-Analysis

**DOI:** 10.31557/APJCP.2020.21.12.3677

**Published:** 2020-12

**Authors:** Thaís Torres Barros Dutra, Thâmara Manoela Marinho Bezerra, Ealber Carvalho Macêdo Luna, Francisco Samuel Rodrigues Carvalho, Filipe Nobre Chaves, Paulo Goberlânio de Barros Silva, Fábio Wildson Gurgel Costa, Karuza Maria Alves Pereira

**Affiliations:** 1 *Department of Clinical Dentistry, Faculty of Pharmacy and Dentistry and Nursing, Federal University of Ceara, Fortaleza, Brazil. *; 2 *School of Dentistry, Federal University of Ceara, Campus Sobral, Sobral, Brazil. *; 3 *Departament of Morphology, School of Medicine, Federal University of Ceará, Brazil. *

**Keywords:** Cancer biomarkers, carcinogenesis, molecular biology

## Abstract

**Background:**

Protocadherins (*PCDHs*) have been reported as tumor suppressor genes, implying that these genes may be involved in tumor suppression in a variety of cancers. However, a thorough understanding of the functions and mechanisms of *PCDHs* remains limited. Our aim was to investigate the methylation profile of *PCDHs* in human malignant neoplasms.

**Methods:**

This systematic review has been recorded in PROSPERO (#42019117844) and conducted according to PRISMA’s checklist; search was conducted in LILACS, PubMed, Science Direct, Scopus, and Web of Science databases, manually, with search queries and without date or language restrictions.

**Results:**

We found 91 articles, of which 26 were used for this meta-analysis and categorized according to the origin of the neoplasia. In total, 3,377 cases were compiled, with* PCDH10, PCDH17*, and *PCDH8* being the most studied; males were 2.22 times more affected than females. Studies have shown significant heterogeneity (p <0.001), with the odds ratio varying between cases and controls [2.20 (95% CI = 1.11– 4.35) to 209.05 (95% CI = 12.64– 2,457.18)], and the value of association between methylation and cancers studied was 26.08 (95% CI = 15.42–44.13).

**Conclusion:**

In this systematic review, we have demonstrated using meta-analysis that PCDHs could emerge as potential tumor suppressor genes and that a significant increase in methylation may be useful for early detection of different cancers. This work may help in the identification of new prognostic biomarkers in malignant neoplasms.

## Introduction

Cadherin is a calcium-dependent adhesion protein that is a member of a large family of cell adhesion molecules. Cadherins can be classified into three groups: classical, desmosomal, and protocadherins *(PCDHs). PCDHs* are predominantly expressed in the nervous system and are primarily involved in the maintenance of brain functions. However, in recent years, it has been revealed in few studies that members of the *PCDH* family may have other functions, including functions in some types of cancer (Narayan et al., 2009; Harada et al., 2015; Keeler et al., 2015; Shi et al., 2015; Deng et al., 2016; Lee et al., 2016; Ye et al., 2017).

*PCDHs* are the largest subgroup of the cadherin superfamily and can be divided into two groups based on their genomic structure: clustered *PCDHs*, constituting gene clusters on a single chromosome, and ungrouped *PCDHs*, spread over different chromosomes (Wu et al., 2017; Zhong et al., 2017). Ungrouped human *PCDH *genes are often located at three chromo-somal loci: 4q28–31, 5q31–33, and 13q21 (Kim et al., 2011).

In contrast to classical cadherin, which establishes strong cell-cell adhesion through homo-philic interactions, *PCDHs* have varied physiological functions, acting both as a mediator of cell-cell adhesion and as a regulator of other molecules (Yagi and Takeichi, 2000; Kim et al., 2011). In addition, according to a study, *PCDHs* located on chromosome 13q21, such as *PCDH8, PCDH9, PCDH17*, and *PCDH20*, may be involved in tumor suppression (Kim et al., 2011). Recently, some *PCDHs* have been reported as tumor suppressor genes, implying that these genes may be involved in tumor suppression in a variety of cancers (Imoto et al., 2006; Narayan et al., 2009; Yu et al., 2009; Hu et al., 2013; Wang et al., 2014; Keller et al., 2015; Shi et al., 2015; Deng et al., 2016; Lee et al., 2016; Ye et al., 2017; Zhong et al., 2017).

A detailed understanding of the functions and mechanisms of* PCDHs* remains limited, and, so far, there has been no systematic literature review in which the underlying mechanisms of *PCDH* activity in carcinogenesis have been discussed. Thus, understanding the participation and actual role of *PCDHs* in important cancer signaling pathways is fundamental for better elucidation of the regulatory and progressing intracellular mechanisms of carcinogenesis. Hence, our aim in this review was to investigate the expression of *PCDHs* in primary malig-nant neoplasms.

## Materials and Methods

This systematic review was conducted to summarize the current knowledge on *PCDH* expression in cancer, according to the Checklist of Preferred Reporting Items for Systematic Re-views and Meta-Analyses (PRISMA) (Additional file 1). The protocol has been registered in the International Prospective Register of Systematic Reviews (PROSPERO) database with the registration number CRD42019117844.

*Information sources and search strategy:*

The studies to be considered for inclusion were identified using a search strategy in each of the following electronic databases: LILACS, PubMed, Science Direct, Scopus, and Web of Science (Online Resource 1) (Additional file 2). A partial search for gray literature was per-formed using Google Scholar, OpenGrey, and ProQuest Dissertations and Theses Global. The gray database and bibliographic search included all articles published until October 21, 2019, without time restriction. Duplicate references were removed using the reference manager software (EndNote^®^, Thomson Reuters). In addition, the reference lists of selected articles were manually selected for possible relevant studies that might have been missed during electronic database search.

*Eligibility Criteria*

Articles were selected without restrictions in year or language of publication; and the inclusion criteria adopted in this review were as follows: laboratory studies involving PCDH analysis in cancer and studies involving humans. The exclusion criteria were as follows: case reports, animal studies, literature reviews, and studies in which the clinical-pathological parameters were not presented.

*Study selection*

The selection of articles was performed in two phases. In phase 1, two reviewers independently (TTBD and EMCL) verified the titles and summaries of all of the identified electronic database citations. A third reviewer was involved as needed to make the final decision (KMAP). Studies that did not meet the inclusion criteria were omitted (Additional file 3). In phase 2, the same selection processes was carried out for full papers to confirm if the articles were suitable, according to inclusion criteria. TTBD and EMCL independently participated in phase 2 as well. The reference list of all of the included articles was reviewed by an examiner; however, both examiners read the selected articles. Any disagreement at any stage was resolved by discussion and mutual agreement.

*Data collection process*

One author (TTBD) collected important information from each article selected. A second reviewer (EMCL) cross-checked the information collected and confirmed its accuracy. Any disagreement between them was resolved by discussion and mutual agreement. For all studies included, the following information was recorded: author(s), year of publication, country, sex, age, smoking status, alcohol consumption pattern, HPV infection, sample size (study group and control group), tissue type (control and cancer), histological type, tumor differentiation, clinical staging, tumor size, tumor volume, lesion location, serum PSA (where applicable), disease progression, margin status, metastasis, biochemical recurrence (where applicable), follow-up time, death rate, survival, methodological design, and main results.

*Risk of bias in individual studies*

The risk of bias analysis of the studies was performed independently by two authors (TTBD and EMCL), in accordance to a questionnaire developed by the Joanna Briggs Institute (2017). The authors rated each item of the questionnaire as “yes”, “no”, “inaccurate”, or “not applicable”. The risk of bias was rated as high (up to 49% yes), moderate (50% to 69% yes), and low (over 70% yes).

*Summary measures and synthesis of results*

The primary outcome for this systematic review was the use of *PCDH *as a biomarker for cancer diagnosis. A secondary outcome would be the role of *PCDH* as a tumor suppressor. Any type of measurement was considered in this review (categorical and continuous variables). Data were tabulated in an Excel® spreadsheet (Microsoft Corporation, Redmond, WA, USA) for relative and absolute frequencies and then exported to the Statistical Package for Social Sciences (version 17, IBM, Armonk, New York, USA) to perform statistical analysis. For meta-analysis, data were exported to MedCalc software for case-control meta-analysis. Der-Simonian Laird analyses were used to calculate the combined random effect odds ratios. A 95% confidence interval was used for all evaluations. A total of 26 articles were included in the meta-analysis, which were also categorized according to the origin of the neoplasia (upper respiratory tract, gastrointestinal tract , and genitourinary tract).

## Results

*Study selection*

**Figure 1:**
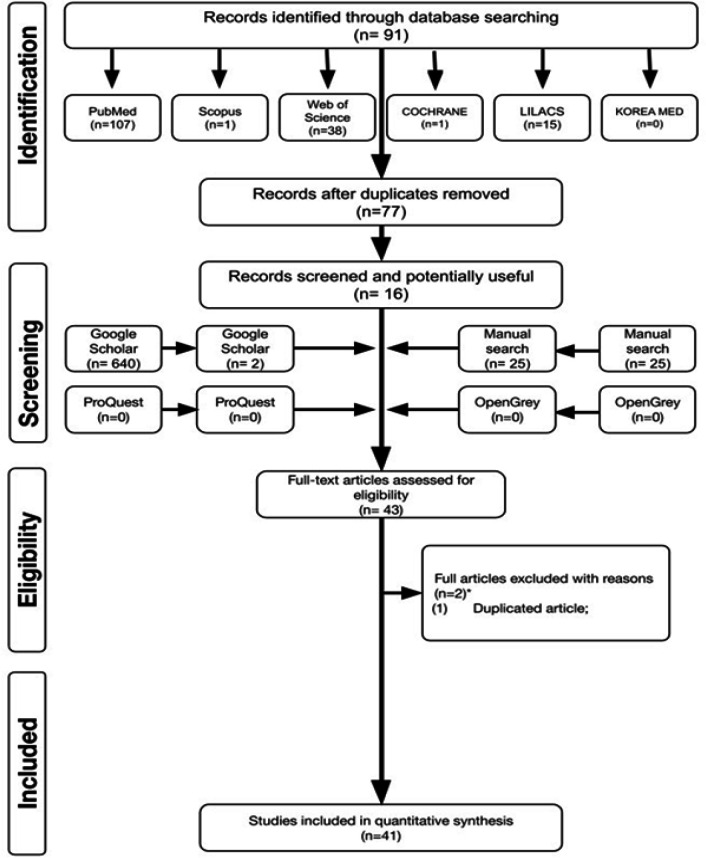
Flow Diagram of PRISMA's Adapted Literature Search and Selection Criteria

At the end of phase 1, we could obtain 91 bibliographic references from the electronic database. However, 14 articles were present in more than one database (duplicate articles), after the removal of which, 77 articles remained for independent analysis by the reviewers. In addition, a manual search was conducted, and we found 25 additional articles from Google Scholar; however, we excluded 2 articles as these were duplicated. A flowchart of the study selection has been presented in Figure 1.

A full-text review was independently conducted by the reviewers, and 41 articles that met the inclusion criteria were selected. The studies that were excluded were as follows: laboratory studies involving *PCDH* analysis in other pathologies; studies on blood, lymphatic, or brain malignant neoplasms; studies that did not use tumor samples for analysis; animal studies; and studies without clinical-pathological parameters described.

*Study characterization*

All of the studies included in this review were published in English language between the years 2006 and 2018 and were conducted in seven different countries, with most of the studies published in China (n = 34), followed by Japan (n = 3), Italy (n = 2), the Netherlands (n = 1), and the Czech Republic (n = 1). A summary of the descriptive characteristics of the included studies has been presented in Table 1.

In total, there were 6,645 cases on malignant neoplasm in the forty-one studies that were selected as sample, and the number of cases in the sample ranged between 2816 and 107,217. The total number of controls were 1,934, and 7 studies reported no control group (Losi et al., 2011; Chen et al., 2015; Harada et al., 2015; Hou et al., 2015; Lv et al., 2015; Wu et al., 2017; Cao et al., 2018).

**Figure 2:**
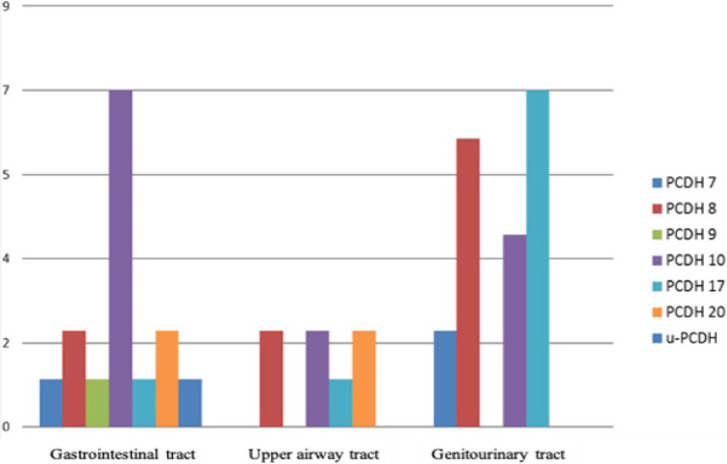
Distribution of the Types of PCDHs Studies among the Categories of Neoplasms (n = 41)

Most of the studies reported genitourinary tract (n = 19; 46.34%) or gastrointestinal tract (n = 15; 36.59%) neoplasms. From the studies in which the patient’s gender was reported, we observed that 74.43% (5,087/6,835) of the patients were male and 25,57% (1,748/6,835) were female, with the average male:female ratio being 2.91. The most-studied *PCDHs *were *PCDH10, PCDH8*, and *PCDH17 *(Figure 2).

*Immunoexpression of PCDHs in malignant neoplasms is negative or low, and methylation is a specific tumor event*

The immunohistochemical profile was evaluated in 13 articles in which negative or low immunoexpression was reported (Haruki et al., 2010; Losi et al., 2011; Ma et al., 2013; Chen et al., 2015; Chen et al., 2015; Harada et al., 2015; Dang et al., 2016; Lin et al., 2016; Zhang et al., 2016; Chen et al., 2017; Wu et al., 2017; Cao et al., 2018; Li et al., 2018). The methylation profile of PCDHs in malignant neoplasms was evaluated in 33 studies (Imoto et al., 2006; Yu et al., 2009; Haruki et al., 2010; Yu et al., 2010; He et al., 2012; Zhang et al., 2012; Lin et al., 2012; Beukers et al., 2013; Danese et al., 2013; Fang et al., 2013; Lin et al., 2013; Tang et al., 2013; Deng et al 2014; Wang et al., 2014; Lin et al., 2014; Lin et al., 2014; Lin et al., 2014; Luo et al., 2014; Niu et al., 2014; Wang et al., 2014; Chen et al., 2015; Chen et al., 2015; Harada et al., 2015; Hou et al., 2015; Lin et al., 2015; Lin et al., 2015; Lv et al., 2015; Deng et al., 2016; Lin et al., 2016; Zhang et al., 2016; Lin et al., 2017; Lin et al., 2017; Baranova et al., 2018; Li et al., 2018), and in 7 studies (Imoto et al., 2006; Beukers et al., 2013; Chen et al., 2015; Harada et al., 2015; Hou et al., 2015; Lv et al., 2015; Lin et al., 2016) the values for controls were not reported. Overall, methylation occurred more frequently in the cases than in the controls.

*Malignant neoplasms show significant increase in methylation*

**Figure 3:**
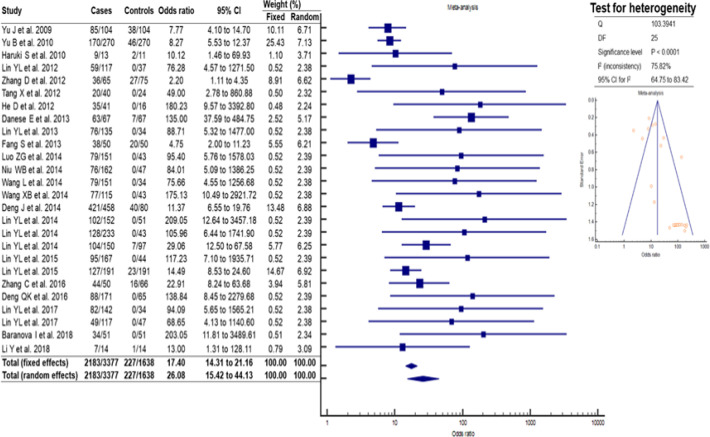
Methylation Profile in Human Malignant Neoplasms (n = 26)

**Figure 4:**
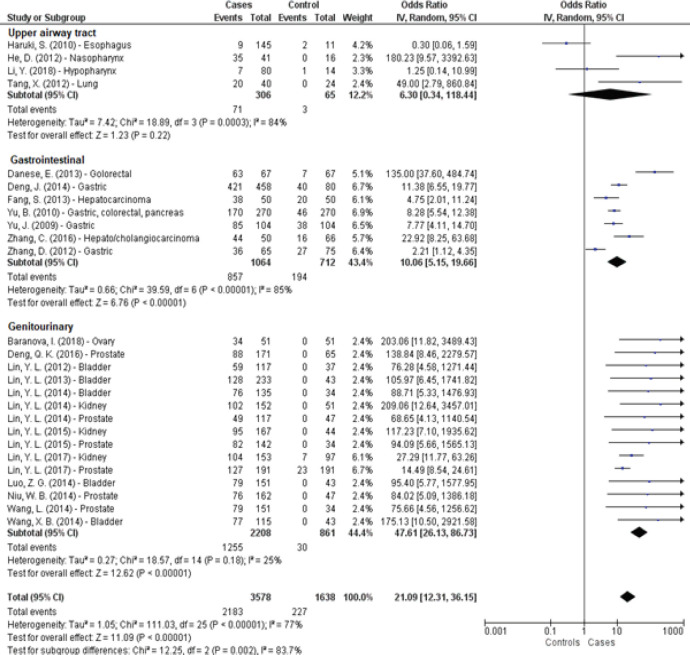
Methylation Profile in Human Malignant Neoplasms (n = 26) Categorized by Group

In total, 26 articles were included in this meta-analysis, which were also categorized according to the origin of neoplasia, and 3,377 cases and 1,638 controls were compiled. In the studies, we observed that there was significant heterogeneity (p <0.001), with a fairly variable odds ratio between cases and controls [2.20 (95% CI = 1.11. 4.35) to 209.05 (95% CI = 12.64. 2.457.18)], and the value of association between methylation and the cancers studied was found to be 26.08 (95% CI 15.42. 44.13) (Figure 3). Depending on the site of origin of neoplasm, the tumors of the upper respiratory tract and gastrointestinal tract (GIT) showed significant heterogeneity, p = 0.0003 and p = 0.00001, respectively, while the tumors of the genitourinary tract showed no heterogeneity (Figure 4).

*Risk of bias in individual studies*

A summary of the risk of bias of the 26 studies included in this meta-analysis has been presented in Table 2. Three studies were classified as having a moderate risk of bias, while in other studies the risk of bias was low. The criteria for inclusion in the sample were clearly defined (Table 2) in five studies (Yu et al., 2009; Haruki et al., 2010; Lin et al., 2012; Danese et al., 2013; Lin et al., 2013). Confounding factors were uncertain, and strategies for dealing with these factors were not applied in all studies. The other items were entirely scored as “yes”

**Table 1:** Summary of Descriptive Characteristics of the Included Studies (n = 41)

Author (year), Country	Localization of Malignant Neoplasia	N Case	N Controls	Gender	PCDH	Method Analyse	Conclusions
Baranova et al. (2018), Czech Republic	Ovary	51	35	NI	PCDH17	Methylation	PCDH17 methylation was detected in almost 70% of group case and gene expression analysis revealed decreased expression in all of the tumor samples in comparison to the control ones. Statistically significant negative correlation was found between methylation and levels of expression suggesting potentially methylation-based silencing.
Bing et al. (2018), China	Hepatic	109	109	F: 51 M: 58	PCDH10	Gene expression	PCDH10 expression was lower in tumor tissues than that in adjacent nontumor tissues. Kaplan–Meier curves showed that patients with lower PCDH10 expression had a worse overall survival. Moreover, PCDH10 expression level was associated tumor size, tumor node metastasis stage, smoking status and drinking status.
Cao et al. (2018), China	Ovary	68	0	NI	PCDH8	Immunohistochemistry and others	Protocaderin-8 can be considered as a tumor suppressor and play a crucial role in the progression of ovarian cancer.
Li et al. (2018), China	Hypopharynx	80	80	F: 2 M: 78	PCDH8	Immunohistochemistry, Methylation and others	PCDH8 may serve as a useful prognostic biomarker and potential therapeutic target for patients with hypopharyngeal carcinoma.
Chen et al. (2017), China	Gastric	119	75	F*: 21 (17.6%)M*: 98 (82.4%)	PCDH7	Immunohistochemistry and others	Low expression and decreased PCDH7 may promote cell migration and invasion by inhibiting E-cadherin expression.
Lin et al. (2017), China	Prostate	117	47	M: 117	PCDH8	Methylation	PCDH8 was methylated in serum samples of case than in controls. This methylation was correlated with advanced clinical stage, higher level of preoperative PSA, and positive lymph node metastasis. Moreover, patients with PCDH8 methylation had worse BCR-free survival than patients without.
Lin et al. (2017), China	Kidney	142	34	F: 45 M: 97	PCDH17	Methylation	PCDH17 was more methylated in case than in controls and methylation in serum was correlated with advanced stage, higher grade, lymph node metastasis and tumor progression. In addition, patients with methylated PCDH17 had shorter progression-free survival and overall survival than patients without, and methylation in serum was an independent prognostic factor for worse progression-free survival and overall survival of patients.
Wu et al. (2017), China.	Hepatic	317	0	F: 35 (11%)M: 282 (89%)	PCDH20	Immunohistochemistry	Decreased expression of protocadherin 20 was observed in patients and was an independent risk factor for mortality.
Deng et al. (2016), China	Prostate	171	65	M*: 236	PCDH10	Methylation	PCDH10 methylation was significantly associated with higher preoperative PSA level, advanced clinical stage, higher Gleason score, lymph node metastasis and BCR. In addition, patients with methylated PCDH10 had shorter BCR-free survival and overall survival. The methylation in serum is an independent predictor of worse BCR-free survival and overall survival.
Lin et al. (2016), China	Bladder (non invasive muscle)	199	25	F: 61 (30.7%)M: 138 (69.3%)	PCDH7	Immunohistochemistry	PCDH7 expression was decreased in invasive non-muscular bladder cancer tissues and low PCDH7 expression was associated with high pathological grade, relapse, and tumor progression. In addition, low expression is an independent prognostic factor for patient outcome.
Zhang et al. (2016), China	Hepatic	50	50	F*: 39 M*: 11	PCDH8	Immunohistochemistry and Methylation	PCDH8 is often inactivated by promoter methylation in liver cancer and can serve as a valuable diagnostic biomarker for early detection and for predicting an unfavorable clinical feature.
Chen et al. (2015), China	Nasopharyngeal	51	13	F*: 12 M*: 39	PCDH20	Immunohistochemistry, Methylation and others	PCDH20 can inhibit cell proliferation, migration and invasion by antagonizing the Wnt/β-catenin and EMT signaling pathway in nasopharyngeal cancer.
Chen et al. (2015), China	Gastric	1 072	0	F: 315 (29.4%)M: 757 (70.6%)	PCDH9	Immunohistochemistry, Methylation and others	Decreased expression of PCDH9 is frequent in metastases of human gastric cancer and its expression is an independent prognostic factor.
Dang et al. (2015), China	Hepatic	86	78	F: 22 M: 64	PCDH17	Immunohistochemistry and others	PCDH-17 expression was clinically correlated with overall prognosis as well as metas- tasis in vivo and inhibit metastasis via EGFR/MEK/ERK signaling pathway ex vivo.
Harada et al. (2015), China	Lung (non-small cells)	109	0	F: 42 M: 67	PCDH10	Immunohistochemistry and Methylation	PCDH10 promoter methylation plays a significant role in the progression of non-small cell lung cancer and may be a promising prognostic marker for patients with curatively resected pathological stage I.
Author (year), Country	Localization of Malignant Neoplasia	N Case	N Controls	Gender	PCDH	Method Analyse	Conclusions
Hou et al. (2015), Japan	Gastric	471	0	F: 118 (25.1%)M: 353 (74.9%)	PCDH10	Methylation	Current findings suggest that the count of hypermethylated CpG sites from the PCDH10 DNA promoter to assess the prognosis of gastric cancer.s
Lin et al. (2015), China	Prostate	167	44	M: 211	PCDH17	Methylation	PCDH17 methylation was associated with advanced pathological stage, higher Gleason score, high- er preoperative PSA levels, BCR, and shorter BCR-free survival.
Lin et al. (2015), China	Kidney	191	191	F*: 77 (40.3%)M*: 114 (59.7%)	PCDH17	Methylation	PCDH17 methylation is significantly correlated with advanced stage, higher grade, and lymph node metastasis. Moreover, it is an independent prognostic factor for progression-free survival and overall survival of patients.
Lv et al. (2015), China	Hepatic	107	0	F: 35 M: 72	PCDH20	Methylation and others	PCDH20 can inhibit cell proliferation and cell migration, through antagonizing Wnt/b-catenin signalling pathway
Deng et al. (2014), China	Gastric	458	25	F*: 145 (31.6%)M*: 313 (68.34%)	PCDH10	Methylation and others	Protocadherin-10 promoter methylation was more in case and was associated with poorer survival.
Lin et al. (2014), China	Prostate	152	51	M: 203	PCDH17	Methylation	PCDH17 methylation occurred in prostate cancer and was associated with higher pathological Gleason score, advanced pathological stage, higher level of preoperative PSA, positive angiolymphatic invasion, positive lymph node metastasis, and BCR. In addition, methylation was an independent predictor of poor BCR-free survival and overall survival for patients with prostate cancer.
Lin et al. (2014), China	Kidney	153	97	F*: 51 (33.3%)M*: 102 (66.7%)	PCDH8	Methylation	PCDH8 methylation was more frequent in tumor tissues and was significantly correlated with advanced clinical stage, higher grade , and lymph node metastasis. In addition, methylation was independently associated with poor progression-free survival.
Lin et al. (2014), China	Bladder (non invasive muscle)	233	43	F*: 72 M*: 161	PCDH8	Methylation	PCDH8 methylation occurred in tumor tissues and was correlated with advanced stage, high grade, larger tumor size, tumor recurrence and progression. The patients with PCDH8 methylated have shorter recurrence-free survival, progression-free survival and five-year overall survival.
Luo et al. (2014), China	Bladder	151	43	F: 56 M: 138	PCDH17	Methylation	PCDH17 promoter methylation was detected in 52.3% of patients with bladder cancer and was associated with larger tumour diameter, high grade and advanced stage. Patients with PCDH17 promoter methylation had significantly shorter overall survival than those with unmethylated PCDH17 promoter.
Niu et al. (2014), China	Prostate	162	47	M: 209	PCDH8	Methylation	PCDH8 methylation occurred in tumor tissues and was associated with advanced pathologic stage, higher level of preoperative PSA, higher Gleason score, positive lymph node metastasis, and biochemical recurrence. The patients with methylated have shorter BCR-free survival time.
Wang et al. (2014), China	Prostate	151	34	M: 185	PCDH10	Methylation	PCDH10 methylation was more in tumor tissue and was associated with higher preoperative PSA level, higher Gleason Score, advanced clinical stage, lymph node metastasis, angiolymphatic invasion, biochemical recurrence and may be used as an independent predictor of BCR-free survival.
Wang et al. (2014), China	Bladder	115	43	F: 45 M: 113	PCDH17	Methylation	Methylation of the PCDH17 promoter was detected in tumor tissueand was associated with high cancer grade, advanced cancer stage, large tumour diameter and tumour recurrence. Methylation was also associated with significantly shorter survival time.
Beukers et al. (2013), Netherlands	Bladder	167	35	F*: 36 (22%)M*: 131 (78%)	PCDH7	Methylation	PCDH7 showed high methylation ratios in all age categories and could therefore play an important role in early urothelial carcinogenesis.
Danese et al. (2013), Italy	Colorectal	67	67**	F: 22 (34.9%)M: 41 (65.1%)	PCDH10	Methylation	PCDH10 methylation detected in plasma increased with increasing methylation rate in tumor tissues only in early stage cancers, while this correlation was apparently lost in advanced stages.
Fang et al. (2013), China	Hepatic	50	50**	F: 16 M: 34	PCDH10	Methylation and others	PCDH10 methylation was detected in tumor tissues compared. There were correlations between methylation status of and tumor size, serum AFP levels, metastasis or TNM staging.
Lin et al. (2013), China	Bladder	135	34	F: 49 M: 120	PCDH8	Methylation	PCDH8 promoter methylation was detected in tumor tissue and was associated with advanced stage, high grade, tumour recurrence, larger tumour diameter and nonpapillary morphology. In addition, methylation was associated with significantly shorter survival time and was an independent predictor of overall survival.
Author (year), Country	Localization of Malignant Neoplasia	N Case	N Controls	Gender	PCDH	Method Analyse	Conclusions
Ma et al. (2013), China	Bladder	105	33	F: 38 M: 100	PCDH10	Immunohistochemistry	Downregulated PCDH10 levels correlated with malignant behaviour and poor overall survival in patients with bladder cancer. Downregulated
He et al. (2012), China	Nasopharyngeal	41	16	F: 10 M: 31	PCDH8	Methylation and others	Ectopic expression of PCDH8 in silenced NPC cells significantly inhibited cell colony formation and cell migration. Thus, PCDH8 could be identified as a tumor suppressor in this cancer.
Lin et al. (2012), China	Bladder	117	37	F: 50 M: 104	PCDH10	Methylation	PCDH10 promoter methylation was detected in tumor tissue and was associated with advanced stage, high grade, tumour recurrence and larger tumour size. In addition, methylation was associated with significantly worse survival and was an independent predictor of overall survival.
Tang et al. (2012), China	Lung	40	24	F*: 12 M*: 28	PCDH10	Methylation and others	PCDH10 was downregulated in tumor tissues and methylation of was observed % tumor tissues but not in tumor-adjacent or normal tissues. Ectopic expression of PCDH10 in silenced cells can reduce lung cancer cell proliferation and migration.
Zhang et al. (2012), China	Gastric	65	10	F*: 23 M*: 42	PCDH8	Methylation and others	PCDH8 methylation was observed in alls cell lines and 55.38% of the primary tumor, but not in normal gastric mucosa, and was associated with lymph node metastasis.
Losi et al. (2011), Italy	Colorectal	28	0	F: 13 M: 15	u-PCDH	Immunohistochemistry and others	Down regulation of μ-protocadherin expression is a common event in colorectal carcinogenesis and may therefore play an important role in this pathological process.
Haruki et al. (2010), Japan	Esophageal	145	13**	F*: 16 M*: 129	PCDH17	Immunohistochemistry, Methylation and others	Silencing of PCDH17 expression through hypermethylation of the promoter or other mechanisms leads to loss of its tumour-suppressive activity.
Yu et al. (2010), China	Gastric, Colorectal, Pancreatic	270	270**	NI	PCDH10	Methylation	PCDH10 methylation was higher in precancerous lesions than in chronic gastritis samples and Kaplan–Meier survival curves showed that PCDH10 methylation was associated signif- icantly with shortened survival in stage I–III gastric cancer patients.
Yu et al. (2009), China	Gastric	104	104**	F*: 45 M*: 57	PCDH10	Methylation and others	A PCDH10 é um supressor de tumor gástrico; sua metilação nas fases iniciais da carcinogênese gástrica é um fator prognóstico independente.
Imoto et al. (2006), Japan	Lung (non-small cells)	59	12	F: 20 M: 39	PCDH20	Methylation and others	Methylation of this PCDH20 promoter was observed in primary tumor and was associated with a shorter overall survival. Moreover, the PCDH20 methylation status was an independent prognosticato

* Data entered for control purposes only; **, peri-lesional tissue; NI, not informed; PCDH, Protocadherin; F, female; M, male; PSA, prostate- specific antigen; BCR, biochemical recurrence; CpG, site of cytosine and guanine; AFT, Alpha Fetoprotein; TNM, Classification of Malignant Tumors.

**Table 2:** Summary of Risk Assessment of Bias in Individual Studies (n = 26)

Author (year), Country	Risk of bias in individual studies
Baranova et al. (2018), Czech Republic	Low
Li et al. (2018), China	Low
Lin et al. (2017), China	Low
Lin et al. (2017), China	Low
Zhang et al. (2016), China	Moderate
Deng et al.(2016), China	Low
Lin et al. (2015), China	Low
Lin et al. (2015), China	Low
Deng et al. (2014), China	Low
Lin et al. (2014), China	Low
Lin et al. (2014), China	Low
Lin et al. (2014), China	Low
Luo et al. (2014), China	Low
Niu et al. (2014), China	Low
Wang et al. (2014), China	Low
Wang et al. (2014), China	Low
Danese et al. (2013), Italy	Low
Fang (2013), China	Low
Lin (2013), China	Low
Zhang et al. (2012), China	Low
He et al.(2012), China	Low
Lin et al. (2012), China	Low
Tang et al.(2012), China	Moderate
Haruki et al. (2010), Japan	Low
Yu et al. (2010), China	Low
Yu et al. (2009), China	Moderate

## Discussion

*PCDHs* are proteins that play an important role in establishing specific cell connections and developing tumors (Narayan et al., 2009; Harada et al., 2015; Keeler et al., 2015; Shi et al., 2015; Deng et al., 2016; Lee et al., 2016; Ye et al., 2017). Ours is the first systematic review and meta-analysis, in which we investigated the PCDH activity in carcinogenesis, in the available literature. Seven types of PCDHs were analyzed: *PCDH7, PCDH8, PCDH9, PCDH10, PCDH17, PCDH20*, and *u-PCDH. PCDH10, PCDH17*, and *PCDH8* were the most researched and were located at the chromosomal loci 4q28.3, 13q14.3, and 4p15, respectively.

*PCDH10* is a non-fragmented delta2-PCDH (subgroup of the delta-PCDH subfamily), with a unique cytoplasmic domain (Hirano et al., 1999); and unlike other members of the *PCDH *family, *PCDH10* was found to exhibit widespread expression (Wolverton and Lalande, 2001). *PCDH8 *and* PCDH17*, in contrast, may have multiple tumor suppression functions, such as involvement in cell-cell adhesion, signal transduction, and growth control, although the exact function of both the *PCDHs *is poorly understood. *PCDHs* are often inactivated by DNA methylation in different neoplasms and function as tumor suppressors. In addition, it was observed that the immunoreactivity of *PCDHs* was downregulated in specimens (Ma et al., 2013; Zhang et al., 2016; Cao et al., 2018; Li et al., 2018); and protein expression was re-duced in methylated tumors, in comparison to non-cancerous tissues or tissues in which there was no methylation (Harada et al., 2015).

Lung cancer accounts for 13% of new cancer cases and is the leading cause of cancer mortality, representing the highest age-standardized mortality rate (26.6 deaths per 100,000 popula-tion) (GBD 2015 Mortality and Causes of Death Collaborators, 2016). Hypopharyngeal carci-noma (HFC) is predominantly squamous cell carcinoma and is the reason for about 5% of head and neck cancers. Nasopharyngeal carcinoma (NFC) is a highly malignant disease, which is more prevalent in southern China, with the overall survival period being 5 years in 70% of the patients afftected (Chou et al., 2008). The evaluation of the role of *PCDHs *in this group of neoplasms was performed and reported in seven studies (16.3%).

*PCDH8* functions as a tumor suppressor (Li et al., 2018). PCDH8 methylation was detected in tumor tissues and not in non-tumor tissues (He et al., 2012; Li et al., 2018). *PCDH8* expression was dysregulated or completely silenced in HFC tumor tissues (Li et al., 2018) and NFC cell lines (He et al., 2012), and the low expression was correlated to the advanced pathological stage of HFC. 

*PCDH10* hypermethylation was detected in lung cancer tissues; however, no association was found between the clinicopathological features, except for the smoking pattern (Tang et al., 2013). In addition, it was observed that there was no difference in recurrence pattern in pa-tients (with or without *PCDH10* methylation) (Harada et al., 2015).

*PCDH20* expression was detected in normal lung and nasopharyngeal tissues, but was silenced or downregulated in non-small cell lung cancer and nasopharyngeal cell lines (Imoto et al., 2006; Chen et al., 2015); the same was also observed in nasopharyngeal carcinoma tissues (Chen et al., 2015). However,* PCDH20* methylation was not associated with the clinicopatho-logical features in NFC (Chen et al., 2015), but was associated with the clinical outcome in severe lung cancer, indicating that the inactivation of PCDH20 could occur regardless of the stage of cancer and that malignancy progression may be involved in this fact (Imoto et al., 2006). Thus, *PCDH20* may have several tumor suppression functions, potentially contributing to tumor growth control, signal transduction, and cell-cell adhesion (Chen et al., 2015).

From this meta-analysis, we observed that the combined odds ratio in upper airway tract tumors of 6.3 (95% CI = 0.34–118.44) for *PCDH* methylation and that these proteins could be biomarkers for early detection of tumors. However, the evidence is not strong because of the heterogeneity in results. Thus, further studies are needed to confirm the effectiveness of using *PCDH* as a biomarker of the tumors discussed herein.

Digestive tract tumors represent a large fraction of human cancers and can occur anywhere in the gastrointestinal tract, but the most common sites are the colon and the rectum. The role of *PCDHs* in this group of neoplasms has been evaluated in fifteen studies, and the most re-searched are *PCDH7, PCDH8, PCDH9*, and PCH10.

Genetic deletion is pivotal for *PCDH10* inactivation in colorectal cancer (CRC), and *PCDH10* silencing or downregulation occurs more frequently in tumor cell lines and primary tumors compared to normal mucosa (Jao et al., 2014). Allelic loss of *PCDH10* is also associated with the progression of clinical staging and distant metastasis. Additionally, this type of loss is an independent prognostic factor for predicting poor survival in patients with CRC. Thus, *PCDH *acts as a tumor suppressor in CRC and plays a role in the restriction of liver metastasis (Jao et al., 2014); *PCDH10* methylation can be detected in patient’s blood circulation (Danese et al., 2013). 

The immunoexpression of u-PCDH is silenced in CRC and its downregulation occurs in both tumor samples and cell lines (Losi et al., 2010). *u-PCDH* has also been shown to retain β-catenin in the cell membrane of normal colon enterocytes, which implies that the release of β-catenin from this site and translocation to the nucleus in tumor cells has occurred (Losi et al., 2010).

Reduced *PCDH17* expression was observed in esophageal squamous cell carcinoma and was closely linked to hypermethylation in the CpG-rich region (Haruki et al., 2010). However, other mechanisms, including post-translational modification, may contribute to this reduction (Haruki et al., 2010). Poorly differentiated tumors are negative for *PCDH17.* In addition, in non-neoplastic esophageal epithelia, *PCDH17* was expressed in the spinous cell layers, implying that this fact may be dependent on the status of cell differentiation and that the silenced expression acts on the de-differentiation of esophageal neoplasms (Haruki et al., 2010). 

Gastric cancer (GC) is the fourth most common gastrointestinal tumor and presents a high mortality rate (Isomoto et al., 2008). In this cancer, *PCDH7 *immunoexpression showed a gradual reduction in the normal tissue to intraepithelial neoplasia and GC, and an even lower immunoexpression in CG lymph node metastasis (Chen et al., 2017). This immunoexpression was inversely or negatively correlated with Lauren’s classification, lymph node metastases, and TNM stage (Chen et al., 2017). This demonstrates that *PCDH7* suppresses GC progression (Chen et al., 2017).

It has also been observed that the loss of expression and high methylation of the* PCDH8* promoter have been detected in the tumor cells of GC. *PCDH8* methylation was also found in peri-lesional tissues, but not in normal gastric tissue, and was associated with lymph node metastasis (Zhang et al., 2012). Loss of *PCDH9* expression is associated with epithelial dif-ferentiation and cell metastasis in gastric cancer (Chen et al., 2015).

There is a significant correlation between methylation and loss of* PCDH10* expression in GC (Yu et al., 2010), and it is silenced or downregulated in GC cell lines. This is the major regulatory mechanism of *PCDH10* inactivation (Yu et al., 2009; Deng et al., 2014). However, there was no association between staging and methylation, which may occur because *PCDH10* methylation is supposed to be the cause rather than the result of carcinogenesis. This suggests that *PCDH10* methylation can be used as a marker for early stage cancer diagnosis (Yu et al., 2010), and the counting of methylated CpG islands has clinical prognostic assessment applicability (Deng et al., 2014; Hou et al., 2015).

Liver cancer is the sixth most commonly diagnosed cancer, the fourth leading cause of cancer death worldwide in 2018, and is most prevalent cancer in Africa and Asia (Bray et al., 2018). *PCDH10, PCDH17* and *PCDH20 *expression levels were downregulated in this neoplasm.

Low *PCDH10* expression correlates with tumor size, TNM stage, smoking status, and alcohol consumption pattern (Bing et al., 2019). The ectopic expression of *PCDH10*, when silenced by methylation, can suppress tumor cell growth, migration, invasion, and colony formation (Fang et al., 2013). In addition, *PCDH20* can inhibit metastasis by preventing cell migration (Lv et al. 2015), because it is a sensitive clinical parameter to predict survival in indolent and early stage cases (Wu et al., 2017). 

The *PCDH17* knockout can inhibit the proliferation, migration, and invasion of hepatocarci-noma cells through the overactivation of the EGFR/MEK/ERK signaling pathway (Dang et al., 2016). PCDH8 methylation correlates with alpha-fetoprotein (AFP) levels in patients with AFP levels >50 ng/mL (Zhang et al., 2016). Thus, methylation is the main mechanism of loss of expression in hepatocarcinoma (Zhang et al., 2016).

Thus, we observed from the studies significant heterogeneity (p <0.001), the value of odds of association between methylation and cancer being 10.06 (95% CI = 5.15–19.66). Thus, PCDHs could be biomarkers for the early detection of GIT cancers. However, the evidence is not strong because to the results, for the reason that the results were not sufficiently homogeneous. This fact can be explained by the heterogeneity of the lesions that involve this group of neoplasms with different etiopathogenesis.

Bladder cancer (BC) is a heterogeneous disease, the outcome of which is difficult to predict, and ranks 10th in cancer incidence worldwide (Kaufman et al., 2009; Grossman, 2011; Mossanen and Gore, 2014). BC can be classified into two types, that is, noninvasive muscle BC (NIMBC) and invasive muscle BC (IMBC), based on histopathological and clinical aspects (Sun and Trinh, 2015). In many studies, the role of PCDHs in this type of cancer has been evaluated, and the immunohistochemical and methylation profiles were the adopted methodologies. The immunoreactivity levels of *PCDH7* and *PCDH10* proteins are lower in NIMBC tissues than in normal bladder epithelial tissues (Ma et al., 2013; Lin et al., 2016). Low *PCDH7* immunoexpression has been associated with high pathological grade, recurrence, and tumor progression; this fact is an independent prognostic factor for clinical outcome (Lin et al., 2016) and is associated with BC invasiveness (Ma et al., 2013).

Prostate cancer (PC) is the fifth leading cause of death in men and is a molecularly heterogeneous disease. Therefore, there is no accurate predictive marker for outcome assessment in cancer patients (Jao et al., 2014). Methylation could occur in *PCDH8, PCDH10*, and *PCDH17* in both PC tumor tissue and serum, and is associated with poor prognosis and shorter biochemical relapse-free survival (Losi et al., 2011; Lin et al., 2014; Viu et al., 2014; Wang et al., 2014; Lin et al., 2015; Lin et al., 2017).

Ovarian cancer (OC) is the second most lethal cancer among women worldwide (Siegel et al., 2017), and PCDHs may represent as a possible direction in the search for tumor biomarkers. *PCDH17* methylation is important in OC carcinogenesis (Baranova et al., 2018). In addition, low *PCDH8* immunoexpression in OC tissues predicts a poor prognosis (Cao et al., 2018).

Renal carcinoma (RC) is one of the most commonly diagnosed urinary malignancies, and the most common histological subtype is clear cell RC, accounting for 80% to 90% of cases (Low et al., 2016; Capitanio and Montorsi, 2016). Aberrant methylation of tumor suppressor genes is involved in tumor initiation and progression (Shenoy et al., 2015). The frequency of *PCDH8* and *PCDH17 *methylation is high in this cancer, and the PCDHs are associated with malignancy clinicopathological characteristics and unfavorable prognosis (Lin et al., 2014; Lin et al., 2015). 

In this meta-analysis, we have shown strong evidence for the occurrence of methylation of some PCDHs in the group of neoplasms discussed in this article, and this is because the results were sufficiently homogeneous. Thus, methylation is a specific event for the tumors discussed and can occur in *PCDH7, PCDH8, PCDH10*, and *PCDH17*.

Most of the studies had the subjects and configuration described in detail, reliably measured the results, and used statistical analysis. On the other hand, we did not identify confounding factors, nor did they describe strategies to minimize these factors.

This review has certain limitations that must be taken into consideration. None of the articles reported the sample size calculation; all census-type samples were collected for convenience. Age was reported heterogeneously in the studies, and it was not possible to categorize indi-viduals to verify the most affected age group. There was a heterogeneous geographic distribu-tion of studies, focusing on Asia. In this review, the research was limited to clinical samples and laboratory techniques, and it was not possible to speculate the definitive clinical utility of *PCDHs*. In addition, there was no standardization of the methodologies adopted. In most of the studies, no follow-up of patients with regard to recurrence and disease progression was reported. Finally, the heterogeneity of malignancies and *PCDHs* made it difficult for us to compare the large number of studies included.

In this systematic review, we have demonstrated that *PCDHs *are often silenced by DNA methylation in different malignant neoplasms, of which genitourinary tract tumors are more prevalent. Thus, *PCDHs* could emerge as potential tumor suppressor genes, and a significant increase in methylation may be useful for early detection of different types of cancers. Moreover, the existence of methodologies with great heterogeneity reinforces the importance of studies to evaluate the role of this group of proteins in the carcinogenesis of other neoplasms.
